# Simultaneous Detection and Viral DNA Load Quantification of Different Human Papillomavirus Types in Clinical Specimens by the High Analytical Droplet Digital PCR Method

**DOI:** 10.3389/fmicb.2020.591452

**Published:** 2020-11-19

**Authors:** John Charles Rotondo, Lucia Oton-Gonzalez, Chiara Mazziotta, Carmen Lanzillotti, Maria Rosa Iaquinta, Mauro Tognon, Fernanda Martini

**Affiliations:** Laboratories of Cell Biology and Molecular Genetics, Department of Medical Sciences, University of Ferrara, Ferrara, Italy

**Keywords:** droplet digital PCR, ddPCR, quantitative PCR, human papillomavirus, viral DNA load, cervical intraepithelial neoplasia, infection, CIN 3

## Abstract

Human papillomaviruses (HPVs) are small DNA tumor viruses that mainly infect mucosal epithelia of anogenital and upper respiratory tracts. There has been progressive demand for more analytical assays for HPV DNA quantification. A novel droplet digital PCR (ddPCR) method was developed to simultaneously detect and quantify HPV DNA from different HPV types. DdPCR was initially tested for assay sensitivity, accuracy, specificity as well as intra- and inter-run assay variation employing four recombinant plasmids containing HPV16, HPV18, HPV11, and HPV45 DNAs. The assay was extended to investigate/quantify HPV DNA in Cervical Intraepithelial Neoplasia (CIN, *n* = 45) specimens and human cell lines (*n* = 4). DdPCR and qPCR data from clinical samples were compared. The assay showed high accuracy, sensitivity and specificity, with low intra-/inter- run variations, in detecting/quantifying HPV16/18/11/45 DNAs. HPV DNA was detected in 51.1% (23/45) CIN DNA samples by ddPCR, whereas 40% (18/45) CIN tested HPV-positive by qPCR. Five CIN, tested positive by ddPCR, were found to be negative by qPCR. In CIN specimens, the mean HPV DNA loads determined by ddPCR were 3.81 copy/cell (range 0.002–51.02 copy/cell), whereas 8.04 copy/cell (range 0.003–78.73 copy/cell) by qPCR. DdPCR and qPCR concordantly detected HPV DNA in SiHa, CaSki and Hela cells, whereas HaCaT tested HPV-negative. The correlation between HPV DNA loads simultaneously detected by ddPCR/qPCR in CINs/cell lines was good (*R*^2^ = 0.9706, *p* < 0.0001). Our data indicate that ddPCR is a valuable technique in quantifying HPV DNA load in CIN specimens and human cell lines, thereby improving clinical applications, such as patient management after primary diagnosis of HPV-related lesions with HPV-type specific assays.

## Introduction

Human papillomaviruses (HPVs) are small DNA tumor viruses that mainly infect mucosal epithelia of anogenital and upper respiratory tracts (Tommasino, 2014; Malagutti et al., 2020). HPVs can be divided into high-risk (HR-HPVs) and low-risk (LR-HPVs) groups according to their ability to induce cellular transformation and carcinogenesis. HR-HPVs have been associated with distinct types of anogenital cancers, such as cervical, vulvar, vaginal, penile and anal neoplasia and oropharyngeal tumors (Tommasino, 2014; Preti et al., 2020).

These viruses replicate in the nucleus of the basal layer cells of epithelia. In these cells, the HPV DNA is maintained in an episomal form with a low-copy number (Kurita et al., 2019). However, integration of HPV DNA within the host genome can occur, thereby promoting gene expression changes and carcinogenesis (Tommasino, 2014; Rotondo et al., 2015).

HPV16 and HPV18 types are the most carcinogenic among HR-HPVs. Indeed, these viruses are linked to about 70 and 60% of all uterine cervical cancers and their pre-malignant lesions, the cervical intraepithelial neoplasia (CIN), respectively (Di Luca et al., 1986; Wang et al., 2018). To a lesser extent, other HPVs, including HPV31/52/58, have been found to be associated with cervical cancers (Bruni et al., 2010). Despite the role of HPV in cervical carcinogenesis is well-known (Martini et al., 2004; Comar et al., 2011a; Tommasino, 2014), the current standard screening test for cervical cancer and CIN lesions is a cytological staining-based technique, known as the pap-smear test. A more sensitive technique, which is the HPV-specific qualitative PCR, has been adopted only in certain circumstances (Berkowitz, 2013). So far, cytology-based screening programs have reduced cervical cancer incidence/mortality. However, since the highest impact of this screening protocol has already been reached, in terms of cervical cancer incidence (Berkowitz, 2013), more analytical protocols are needed to facilitate further reduction of this malignancy. For instance, HPV DNA load determination is a helpful predictor marker for CIN lesion recognition (Sundström et al., 2013). Indeed, the detection of a low copy number of HPV DNA may be an indicator of neoplastic transformation, which in turn reflects the risk of CIN onset/progression (Goodman, 2015).

Quantitative real-time PCR (qPCR) has been widely employed due to (i) its use in HPV DNA load quantification, (ii) its broad detection-range of target molecules and (iii) multiplexing potential. However, qPCR has several intrinsic limitations, including: (i) low sensitivity in quantifying the amount of viral DNA when present in a low-copy number (Caraguel et al., 2011); (ii) lack of precision in estimating small differences in copy number among samples (Hindson et al., 2011); (iii) the need for calibration curves, represented by plasmid vectors carrying viral DNAs, thereby increasing the risk of false-positive results (Rotondo et al., 2019). Therefore, there has been progressive demand for more analytical assays for HPV DNA quantification.

Droplet digital PCR (ddPCR) is an innovative technology, which provides an alternative method for nucleic acids quantification (Tagliapietra et al., 2019, 2020; Torreggiani et al., 2019). This technique offers several potential advantages over qPCR. Indeed, ddPCR enables nucleic acids to be quantified in an absolute manner, without using calibration curves along with improved precision and sensitivity at low template concentrations, as well as having a higher detection rate than qPCR (Hindson et al., 2011). In addition, ddPCR provides a more sensitive method in detecting low amounts of nucleic acids than qPCR (Hindson et al., 2011). For these reasons, in recent years, ddPCR has increasingly been used in clinical practice/laboratory analysis as a diagnostic tool for estimating pathogenic DNA loads, including viral DNA loads (Kuypers and Jerome, 2017). This technology is based on limiting the dilution of PCR volume and Poisson statistics. Before PCR amplification, the sample is randomly partitioned into ∼20,000 droplets. Then, the partitioned sample undergoes traditional PCR. After amplification, droplets are classified into those containing the target molecule (positive) and those that do not (negative), by the amplitude of their fluorescence signal (Hindson et al., 2011). One droplet can only potentially contain one target molecule. Due to these characteristics, ddPCR provides a method for analytically quantifying the actual amount of tested DNA, as each droplet is an independent amplification event (Hindson et al., 2011). Each droplet/reaction is independently analyzed to evaluate the amount of target DNA, as a copy number, at single-molecule sensitivity. The absolute copy number quantification of the target nucleic acids is calculated by Poisson statistics (Hindson et al., 2011).

Previous studies reported on HPV DNA detection/quantification employing ddPCR methods (Biron et al., 2016; Jeannot et al., 2016; Lillsunde Larsson and Helenius, 2017). However, these studies are addressed to the identification of a single specific HPV type. Consequently, a large number of experimental runs is required, as HPV type specific primer sets and TaqMan probes are employed in each experiment (Biron et al., 2016; Jeannot et al., 2016; Lillsunde Larsson and Helenius, 2017).

These methodological limitations prompted us to develop a reliable ddPCR method, which allows the simultaneous detection and quantification of DNA sequences from different HPV types. The sensitivity, accuracy, specificity as well as intra- and inter-run assay variation of the ddPCR were initially assessed in HPV DNAs, from HPV16, HPV18, HPV11, and HPV45 types cloned in recombinant plasmids, used as a control (Malagutti et al., 2020). Then, the assay was extended to HPV DNAs, which were the target of the investigation, in different clinical samples, including uterine pre-neoplastic CIN specimens, as well as in human cell lines. Data obtained by ddPCR were compared to qPCR results employing the same clinical samples.

## Materials and Equipment

• pUC19 recombinant plasmid vectors containing the complete genomes of HPV16 (GenBank, accession no. NC_001526.4), HPV18 (GenBank, accession no. GQ180792.1), HPV11 (GenBank, accession no. HE574705.1), and HPV52 (GenBank, accession no. X74481).
• pGEM1 recombinant plasmid vector containing the complete genome of HPV45 (GenBank, accession no. GQ180792.1).
• Restriction enzyme Bam H1 (Thermo Fisher Scientific, Milan, Italy). Catalog number: #ER0051.
• Restriction enzyme *Eco*RI (Thermo Fisher Scientific, Milan, Italy). Catalog number: #ER0271.
• *Bam*HI-Lsp1109I Buffer (10X) (Thermo Fisher Scientific, Milan, Italy). Catalog number: #B57.
• *Eco*RI Buffer (10X) (Thermo Fisher Scientific, Milan, Italy). Catalog number: #B12.
• Cell lines: SiHa (ATCC^®^ HTB-35^TM^), CaSki (ATCC^®^ CRM-CRL-1550^TM^), HeLa (ATCC^®^ CCL-2^TM^) and HaCaT (Elabscience^®^), Catalog number: #EP-CL-0090.
• QIAmp DNA Blood and Tissue Kit (Qiagen, Milan, Italy). Catalog number: #69504.
• Salmon-sperm DNA (Thermo Fisher Scientific, Milan, Italy). Catalog number: #AM9680.
• QX200^TM^ AutoDG Droplet Digital PCR System-Bio-Rad (Bio-Rad, Segrate, Italy). Catalog number: #1864100.
• QX200^TM^ ddPCR^TM^ EvaGreen Supermix (Bio-Rad, Segrate, Italy). Catalog number: #1864035.
• DG32^TM^ Automated Droplet Generator Cartridges (Bio-Rad, Segrate, Italy). Catalog number: #1864108.
• Automated Droplet Generation Oil for EvaGreen (Bio-Rad, Segrate, Italy). Catalog number: #1864112.
• PX1^TM^ PCR Plate Sealer (Bio-Rad, Segrate, Italy). Catalog number: #1814000.
• Thermal cycler SimpliAmp^TM^ (Applied Biosystem, Milan, Italy). Catalog number: #A24811.
• EIF2C1 gene (Bio-Rad, Segrate, Italy). Catalog number: #10031258.
• CFX96 Touch^TM^ Real-Time PCR Detection System (Bio-Rad, Segrate, Italy). Catalog number: #1855196.
• SsoAdvanced^TM^ Universal SYBR^®^ Green Supermix, Bio-Rad (Hercules, CA, United States). Catalog number: #1725271.

## Methods

### HPV Types and HPV Recombinant Plasmids

Three different pUC19 recombinant plasmid vectors containing the complete genomes of HPV16 (GenBank, accession no. NC_001526.4), HPV18 (GenBank, accession no. GQ180792.1), and HPV11 (GenBank, accession no. HE574705.1) along with one pGEM1 recombinant plasmid vector containing the complete genome of HPV45 (GenBank, accession no. GQ180792.1) were employed for the development of the ddPCR assay. These recombinant plasmids were employed as positive controls (Rotondo et al., 2015). In addition, pUC19 recombinant plasmid containing the complete genome of HPV52 (GenBank, accession no. X74481) was employed as negative control. The complete HPV16/18/11 genomes were kindly provided by Prof. Pozzi, Department of Biotechnology, University of Siena, Italy and cloned in pUC19 vectors (American Type Culture Collection ATCC, Manassasas, VA) (Martini et al., 2004; Bononi et al., 2012). The pGEM1_HPV45/pUC19_HPV52 plasmids, which are commercially available, were a kind gift of Prof. Tommasino, Infectious and Cancer Biology group, International Agency for Research on Cancer (IARC), Lyon, France. The pUC19/pGEM1 recombinant plasmids (initial concentration of 10^9^ copy/μL) were serially 10-fold diluted before subsequent ddPCR/qPCR analyses. Linear DNA templates of HPV16/18/11/52 strains were obtained by digestion of the pUC19_HPV16, pUC19_HPV18, pUC19_HPV11, and pUC19_HPV52 recombinant plasmids with Bam H1 (Thermo Fisher Scientific, Milan, Italy), whereas the linearization of HPV45 was obtained by digestion of the pGEM1_HPV45 recombinant plasmid with *Eco*RI (Thermo Fisher Scientific, Milan, Italy). Restriction enzyme digestions were performed in a total reaction mixture of 20 μL, including 2 μL 10 × enzyme buffer, 1 μL of enzyme, 500 ng plasmid DNA, and ddH_2_O up to 20 μL. The mixture was incubated for 2 h at 37°C, followed by 20 min at 80°C. for the enzyme inactivation. Digestions were analyzed on 0.8% agarose gel (Rotondo et al., 2012; Cosenza et al., 2016).

### Clinical Samples, Cell Lines, and DNA Extraction

CIN pre-neoplastic lesion tissues (*n* = 45) were isolated as described (Rotondo et al., 2015). Institutional Review Board (IRB) approval was obtained from University Hospital of Ferrara Ethical Committee (Authorization n. 160986, December 12, 2016). Informed written consents were obtained from patients. The study was performed in accordance with the Declaration of Helsinki (2008). Human cell lines were: (i) SiHa and CaSki, two HPV16-positive uterine cervical cancer cell lines (Ostrowska et al., 2011); (ii) HeLa, a HPV18-positive uterine cervical cancer cell line (Diao et al., 2015); (iii) HaCaT, a HPV-negative self-immortalized keratinocyte cell line derived from human skin, as negative control (Ram et al., 2018). DNA was isolated with the QIAmp DNA Blood and Tissue Kit (Qiagen, Milan, Italy) (Rotondo et al., 2017a). To avoid cross-contaminations during DNA extraction and ddPCR/qPCR procedures, tight precautions were adopted (Contini et al., 2018). In detail, each DNA sample was isolated, and ddPCR/qPCR amplified simultaneously with two different negative controls, i.e., a mock sample lacking DNA (distilled H_2_O) and a salmon sperm DNA specimen, which is a ready-to-use DNA solution, that is used to verify whether cross-contaminations can occur during DNA extraction and molecular analyses (Del Valle et al., 2008; Dumoulin and Hirsch, 2011; Mazzoni et al., 2017b; Rotondo et al., 2017b). No cross-contaminations were determined, as negative controls resulted negative in all experiments. DNA concentration/quality was analyzed by spectrophotometer and by amplifying the β*-globin* gene (Martini et al., 2004; Pancaldi et al., 2009; Rotondo et al., 2015).

### ddPCR Development (or Set-Up)

**FIGURE 1:**
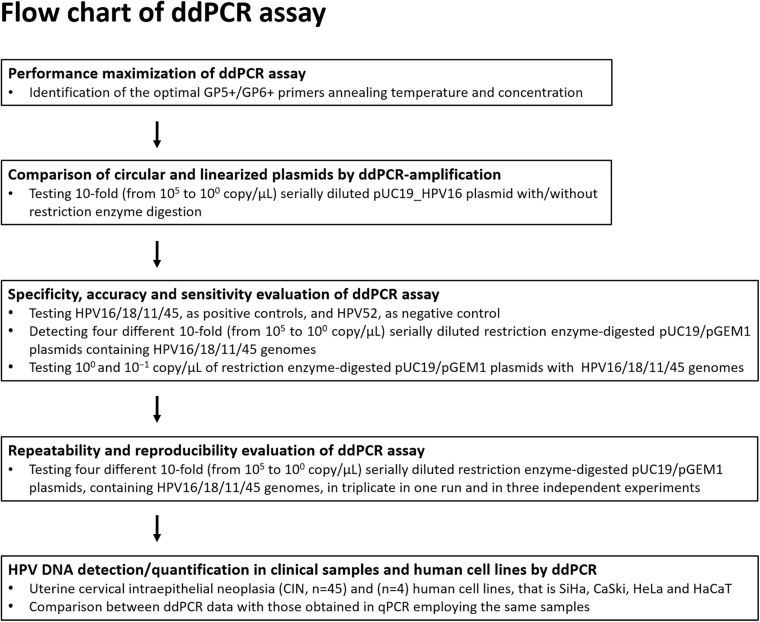
Flow diagram. The ddPCR set up and development for the detection and quantification of HPV16/18/11/45 DNAs from clinical specimens is represented in this diagram.

Different parameters were considered in the ddPCR development (Figure 1). In ddPCR amplification experiments, wide range primers GP5^+^ 5′-TTTGTTACTGTGGTAGATACTAC-3′ and GP6^+^ 5′-GAAAAATAAACTGTAAATCATATTC-3′, which amplify the conserved HPV L1 genomic region of different HPV genotypes, including HPV16/18/11/45 were employed (De Roda Husman et al., 1995; Jacobs et al., 1995; Malagutti et al., 2020). A range of temperatures, from 46 to 60^°^C, with 2°C. intervals, and different primer concentrations, 900, 450, 225, and 112 nM, were tested on 10^3^ copy/μL pUC19_HPV16 (Yang et al., 2017; Liu et al., 2019; Zhang et al., 2019). The ddPCR was then tested on 10-fold serially diluted plasmid pUC19_HPV16 with and without restriction enzyme digestion (Yang et al., 2018). In detail, restriction enzyme digested pUC19_HPV16 plasmid was initially serially diluted to 10^5^, 10^4^, 10^3^, 10^2^, 10^1^, and 10^0^ copy/μL and then tested in ddPCR as template using GP5^+^/GP6^+^ primer set.

In a second phase of the ddPCR, the assay accuracy, specificity and lower detection limit were determined. The accuracy of the ddPCR was assessed by testing scale dilutions of four restriction enzyme-digested pUC19 and pGEM1 recombinant plasmid vectors containing HPV16/18/11 and HPV45 complete genomes, respectively, then comparing the results with the theoretical values. Specifically, the linear range was assessed by scalar dilutions of pUC19_HPV16, pUC19_HPV18, pUC19_HPV11, and pGEM1_HPV45 plasmids from 10^5^ to 10^0^ copies/μl. Each dilution was then detected three times to determine the mean value. Experimental and theoretical values were converted into logarithms and compared (Tagliapietra et al., 2020). The specificity was verified by testing HPV52, which is highly related to HPV16/18/11/45, as negative control. Indeed, it has been reported that its detection using GP5^+^/GP6^+^ primer pair is not reliable (Evans et al., 2005; Chan et al., 2006; Hesselink et al., 2008; Clifford et al., 2016). In detail, 10^5^ copy numbers/reaction linearized pUC19_HPV16, pUC19_HPV18, pUC19_HPV11, and pGEM1_HPV45 were tested together with negative controls, including, 10^5^ copy numbers/reaction linearized pUC19_ HPV52, ssDNA and distilled H_2_O without DNA (Tagliapietra et al., 2020). The sensitivity, as lower detection limit was assessed by testing 10^0^/10^–1^ copy/μL linearized pUC19_HPV16, pUC19_HPV18, pUC19_HPV11, and pGEM1_HPV45 plasmids (Yang et al., 2018; Tagliapietra et al., 2020).

In order to evaluate the repeatability of ddPCR, 10-fold serial dilutions, from 10^5^ to 10^0^ copy/μL, of restriction enzyme-digested linearized pUC19 and pGEM1 plasmids containing the complete genomes of HPV16/18/11/45 were tested in triplicate in one experiment run (Liu et al., 2019). To estimate the reproducibility of ddPCR, these serial dilutions were tested by the ddPCR assay in three independent experiments. The intra- and inter-assay coefficients of variation (CV), plasmid concentrations, as copy number/reaction, and standard deviations (*SD*s) were calculated (Liu et al., 2019).

### ddPCR Experiments

The quantifications of linearized pUC19 and pGEM1 recombinant plasmid vectors containing HPV16/18/11 and HPV45 complete genomes, respectively, and the amount of HPV DNA in template DNA derived from CIN specimens (*n* = 45), SiHa, CaSki, HeLa, and HaCaT cell lines were ddPCR-amplified using the QX200^TM^ Droplet Digital^TM^ PCR System-Bio-Rad (Bio-Rad, Segrate, Italy), according to the manufacturer’s instructions (Tagliapietra et al., 2019, 2020; Mazzoni et al., 2020). The total ddPCR reaction volume of 22 μL contained 11 μL of a 2 × ddPCR super mix (QX200 EvaGreen ddPCR, Bio-Rad, Segrate, Italy), 0.5 μL of each GP5^+^ and GP6^+^ primer, final concentration ranging between 112 and 900 nM each, and 10.5 μL of DNA/ddH_2_O. After equilibrating for 3 min at room temperature, the ddPCR mixture was placed into the DG32 cartridge, and 20 μL of Droplet generation oil for EvaGreen was then added in each well (Bio-Rad, Segrate, Italy). Every sample was partitioned into ∼20,000 stable nano-droplets using an automated droplet generator (Bio-Rad, Segrate, Italy). After processing, the entire prepared droplet emulsion (40 μL) was transferred into a 96 well PCR plate, covered with pierceable foil, heat-sealed using a PX1^*rmTM*^ PCR Plate Sealer (Bio-Rad, Segrate, Italy). Then, the plate was placed in a thermal cycler (SimpliAmp^TM^, Applied Biosystem, Milan, Italy). The cycling conditions were as follows: heat to 95°C for 5 min, followed by 94°C for 30 s and a range of temperature between 46°C and 60°C for 1 min for a total of 45 cycles (at a heating rate of 2°C/s), followed by 5 min at 90°C, ending at 4°C. After PCR, the 96-well PCR plate was placed in the reader. Data were analyzed using the QuantaSoft analysis tool (Bio-Rad, Segrate, Italy). Poisson statistics was used to calculate the absolute concentration of HPV DNAs in each sample (Pinheiro et al., 2012). In order to discriminate positive (blue) and negative (gray) droplets, a threshold line was used. The human EIF2C1 gene (Bio-Rad, Segrate, Italy) was employed to determine the human cell equivalents of each sample under analysis. HPV DNA load was reported as viral copies per human cell equivalents (copy/cell) (Mazzoni et al., 2020).

### qPCR

DNAs from CIN specimens (*n* = 45), SiHa, CaSki, HeLa, and HaCaT cell lines were qPCR-amplified for the detection/quantification of HPV DNA, using the SYBR green chemistry, with the CFX96 Touch^TM^ RT-PCR Detection System (Bio-Rad, Segrate, Italy). Two different recombinant plasmids pUC19 containing the complete genomes of HPV16 and HPV18 were employed as positive controls. DNA samples and plasmid vectors were analyzed in qPCR by using the GP5^+^/GP6^+^ primer set, as reported (de Araujo et al., 2009; Malagutti et al., 2020). A total of 50 ng of human genomic DNA was used in 10μl qPCR reactions, including 2x of the SsoAdvanced Universal SYBR Green Supermix, Bio-Rad (Hercules, CA, United States), with a final concentration of 0.5 μM for each primer. The amplification conditions were: 5 min at 95°C and 40 cycles of: 15 s at 95°C, 30 s at 60°C. HPV16 and HPV18 genotypes were determined in CINs and cell lines, on the basis of differential melting temperature (Tm) obtained in qPCR, as reported (de Araujo et al., 2009). The cellular EIF2C1 gene was used to determine the human cell equivalents of each sample under analysis. In addition, in order to specifically detect HPV16/HPV18 DNAs, two different type-specific primer sets targeting HPV-16/18 genotypes were employed, as reported (Sahiner et al., 2014). HPV DNA loads were reported as viral copies per human cell equivalents (copy/cell). Negative controls were the two DNA extraction negative controls and two additional controls, including HPV free placental DNA and a non-template control (ddH_2_O). Samples were run in triplicate for each qPCR assay. Experiments were run in triplicate by three different operators.

### Statistics

Values were analyzed using the D’Agostino Pearson normality test and then parametric and non-parametric tests were applied according to normal and non-normal variables, respectively, as reported (Campbell and Swinscow, 2009; Nakagawa and Schielzeth, 2010; Mazzoni et al., 2014, 2016). Repeatability (intra-assay variability) and reproducibility (inter-assay variability) of the ddPCR were evaluated by measuring the coefficient of variation (CV), as reported (Brunetto et al., 2014; Liu et al., 2019). In detail, intra-assay variability was determined in triplicate in one experiment run, while inter-assay variability was evaluated by performing 3 independent assays for each HPV type, as reported before for other ddPCR assay set up protocols (Brunetto et al., 2014; Liu et al., 2019). Acceptable CV ranges were considered to be between 0.100 and 0.150 for both intra-assay and inter-assay (U.S. Food and Drug Administration, 2018). Statistical analyses, including linear regression analyses, were carried out using Graph Pad Prism version 4.0 for Windows (Graph Pad, La Jolla, United States) (Tognon et al., 2015; Mazzoni et al., 2017a). Linear regression of correlation coefficienti (*R*^2^) was used to assess ddPCR accuracy and consistency between the ddPCR and qPCR for quantitative HPV DNA detection in samples (Yang et al., 2017, 2018; Dong et al., 2019). *P* < 0.05 were considered statistically significant (Gavanelli et al., 2015; Rotondo et al., 2018).

## Results

### ddPCR Assay Performance Maximization

**FIGURE 2:**
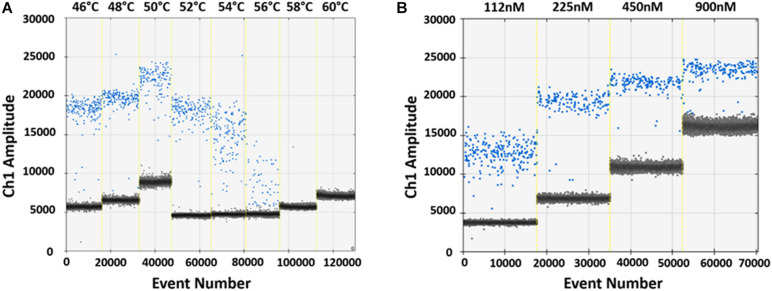
Optimization of ddPCR parameters. **(A)** Optimization of the GP5^+^/GP6^+^ primers annealing temperature using 10^3^ copies/μL pUC19_HPV16 plasmid. Vertical lines represent the gradient of the eight following annealing temperatures: 46, 48, 50, 52, 54, 56, 58, and 60°C. The optimal annealing temperature was 48°C, which resulted in a distinct fluorescence amplitude difference between positive (blue) and negative (gray) droplets. **(B)** Optimization of the GP5^+^/GP6^+^ primers concentration. Optimization was performed using 10^3^ copies/μL pUC19_HPV16 plasmid. Vertical lines represent the different primers concentrations: 112, 225, 450, and 900 nM. The optimal primer concentration resulted 225 nM, based on better amplitude fluorescence spread between positive (blue) and negative (gray) droplets.

The ddPCR assay was initially set up to identify the optimal GP5^+^/GP6^+^ primer annealing temperature and concentration on pUC19_HPV16 plasmid. According to the fluorescence signal, 48°C and 225 nM were selected as the optimal primers annealing temperature and concentration, which resulted in a distinct fluorescence amplitude difference between the positive (blue) and negative (gray) controls (Figure 2).

### Comparison of *Bam*HI Linearized and Circular pUC19_HPV16 Plasmids

**FIGURE 3:**
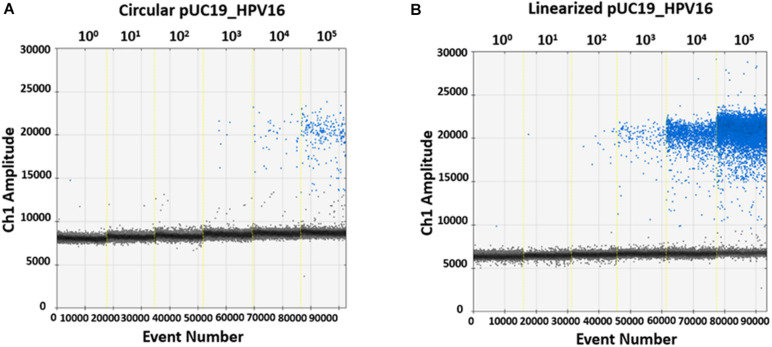
Representative quantification of pUC19_HPV16 plasmid with or without restriction enzyme digestion by ddPCR. **(A)** Copy number of pUC19_HPV16 plasmid without enzyme digestion. The circular pUC19_HPV16 plasmid was serially diluted from 10^5^ to 10^0^ copies/μL and ddPCR amplified. **(B)** Copy number of pUC19_HPV16 plasmid after restriction enzyme digestion. The enzyme-digested linearized pUC19_HPV16 plasmid was serially diluted from 10^5^ to 10^0^ copies/μL and ddPCR amplified. In A and B panels, vertical lines represent the fluorescent amplitude of the different plasmid concentrations: 10^0^, 10^1^, 10^2^, 10^3^, 10^4^, 10^5^ copies/μL.

In order to evaluate ddPCR efficiency on *Bam*HI linearized and circular plasmids, pUC19_HPV16 plasmid with and without restriction enzyme digestion, were 10-fold serially diluted, from 10^5^ to 10^0^ copy/μL, as templates. Then, restriction enzyme-digested linearized and circular pUC19_HPV16 plasmids were ddPCR amplified using the GP5^+^/GP6^+^ primer set (Figures 3A,B). Data indicate that the detected copy numbers of linearized pUC19_HPV16 overlapped with the theoretical value compared to the results of pUC19_HPV16 without restriction enzyme digestion (data not shown). The circular pUC19_HPV16 plasmid resulted as being almost undetectable (Figure 3B). Consistently, the amount of circular pUC19_HPV16 plasmid strongly deviated from the theoretical value (data not shown).

### ddPCR Assay Specificity, Accuracy, and Sensitivity

**FIGURE 4:**
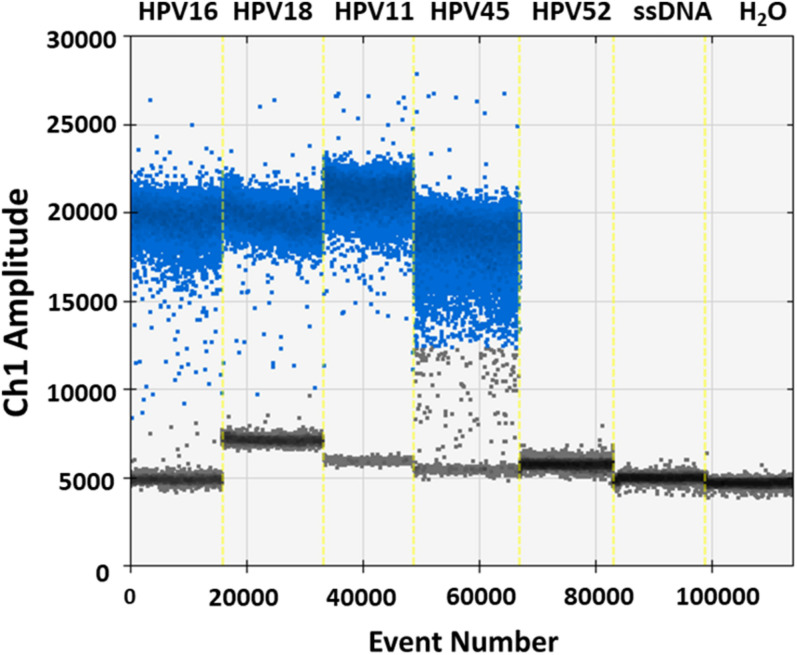
Representative analysis of ddPCR specificity. Vertical lines represent the fluorescent amplitude of pUC19 and pGEM1 plasmids, which contain the complete genomes of HPV16/18/11 and HPV45, respectively, as well as pUC19, which contains the complete genome of HPV52, employed as negative control. Two others negative controls are included: one control contains the salmon sperm DNA (ssDNA), whereas the additional negative control is represented by ddH_2_O without DNA.

**FIGURE 5:**
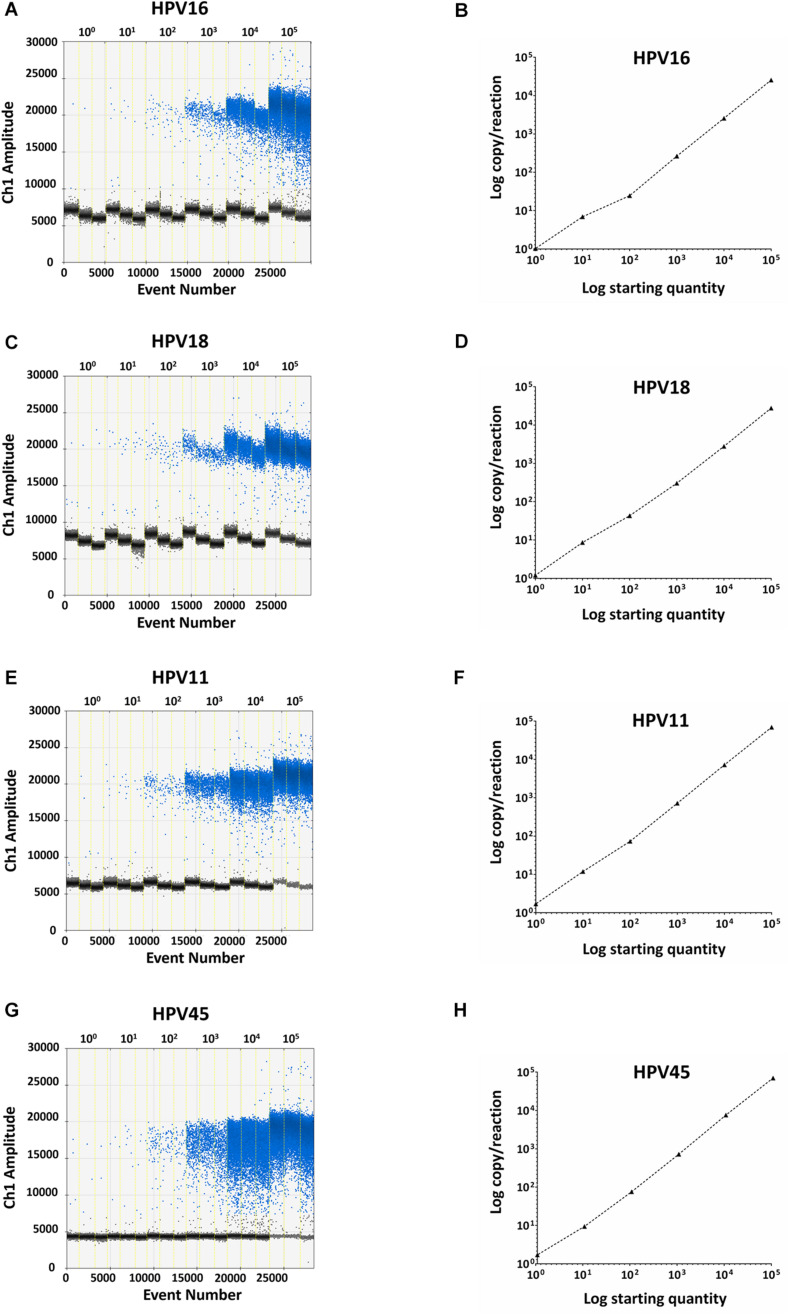
Representative ddPCR amplifications of pUC19/pGEM1 plasmids with HPV16/18/11/45 genomes, together with the correlation between theoretical and detected plasmid concentrations. Ten-fold serial dilutions of pUC19_HPV16 **(A)**, pUC19_HPV18 **(C)**, pUC19_HPV11 **(E)**, pGEM1_HPV45 **(G)** plasmids ranging from 10^0^ to 10^5^ copies/μL are showed. All panels: vertical lines represent the different plasmid concentrations: 10^0^, 10^1^, 10^2^, 10^3^, 10^4^, 10^5^ copies/μL. The correlation was assessed by plotting the log copy/reaction against the log starting concentration of 10-fold serial dilutions of pUC19_HPV16 **(B)**, pUC19_HPV18 **(D)**, pUC19_HPV11 **(H)**, pGEM1_HPV45 **(F)** plasmids. The ddPCR gave high accuracy for pUC19_HPV16, pUC19_HPV18, and pUC19_HPV11, with an *R*^2^ of 1 and a *P* = 0.0001, whereas pGEM1_HPV45 was detected with good accuracy, with an *R*^2^ of 0.9999 and a *p* = 0.0001. Linear regression analysis was performed using GraphPad Prism software.

Specificity analyses were performed employing 10^5^ copy number/reaction of pUC19/pGEM1 plasmids containing HPV16/18/11/45 complete genomes, respectively. A pUC19 plasmid containing the complete HPV52 genome was employed as a negative control. Indeed, it has been reported that the detection of HPV52 using GP5^+^/GP6^+^ primer pair is not reliable (Evans et al., 2005; Chan et al., 2006; Hesselink et al., 2008; Clifford et al., 2016). Results indicate that only plasmids containing HPV16/18/11/45 genomes tested positive, while no positive fluorescence signals were detected when using the plasmid containing HPV52 genome, and the negative controls (Figure 4). DdPCR assay accuracy was verified by detecting 10-fold (from 10^5^ to 10^0^ copy/μL) diluted linearized pUC19/pGEM1 plasmids containing the complete genomes of HPV16/18/11/45 types (Figure 5). Dilutions started at 10^5^ copy/μL, as concentrations ≥10^6^ copy/μL completely saturated the amplitude (data not shown). Each dilution was tested by ddPCR in triplicate. The theoretical/detected values were converted into logarithms and compared. Results indicate a good degree of linear correlation with the dilutions of all tested plasmids, which met the requirements of the ddPCR accuracy. In detail, the ddPCR method presented high accuracy for detecting genomes belonging to HPV16/18/11, with *R*^2^ of 1 and *P* = 0.0001, whereas HPV45 DNA was detected with good accuracy, with *R*^2^ of 0.9999 and *p* = 0.0001 (Figure 5). DdPCR assay sensitivity was assessed, at a lower detection limit, using 10^0^ and 10^–1^ copy number/reaction of linearized pUC19/pGEM1 plasmids containing the complete genomes of HPV16/18/11/45. Plasmids at 10^–1^ copy number/reaction were not detected, whereas the lower detection limit of the ddPCR assay resulted as 10^0^ copy number/reaction for all four plasmids.

### ddPCR Assay Repeatability and Reproducibility

In order to evaluate ddPCR repeatability and reproducibility, linearized pUC19/pGEM1 plasmids containing complete HPV16/18/11/45 genomes were tested in triplicate in one run and in three independent experiments. The intra-assay CV ranged between 0.017 and 1.502 for pUC19_HPV16, 0.020–0.620 for pUC19_HPV18, 0.011–0.633 for pUC19_HPV11, and 0.002–0.733 for pGEM1_HPV45 (Table 1). The inter-assay CV ranged between 0.031 and 0.260 for pUC19_HPV16, 0.040-0.291 for pUC19_HPV18, 0.038–0.721 for pUC19_HPV11 and 0.056–0.572 for pGEM1_HPV45 (Table 1).

**TABLE 1:** Repeatability and reproducibility of the droplet digital PCR assay for HPV16/18/11/45.

	**Intra-assay variation**			**Inter-assay variation**		
**Concentrations**	**Mean**	***SD***	**CV**	**Mean**	***SD***	**CV**
**(copies/μL)**	**(copies/reaction)**			**(copies/reaction)**		
**HPV16**						
10^5^	23,437.3	404.5	0.017	25,352.24	2,405.91	0.095
10^4^	2,434.7	125.1	0.051	2,559.96	146.04	0.057
10^3^	250.9	11.3	0.045	264.07	17.21	0.065
10^2^	27.1	1.6	0.057	24.54	0.77	0.031
10^1^	6.5	9.7	1.502	6.93	1.80	0.260
1	1.0	0.9	0.933	1.03	0.12	0.112
**HPV18**						
10^5^	25,234.0	986.57	0.039	27,522.0	2,002.0	0.073
10^4^	2,757.3	127.01	0.046	2,781.8	133.7	0.048
10^3^	316.8	6.22	0.020	302.9	12.1	0.040
10^2^	51.7	7.78	0.150	42.9	12.5	0.291
10^1^	9.3	1.02	0.109	8.6	1.8	0.205
1	1.2	0.77	0.620	1.2	0.1	0.092
**HPV11**						
10^5^	70,026.7	998.7	0.011	69,228.9	2,622.6	0.038
10^4^	7,440.0	487.9	0.059	7,249.7	315.9	0.044
10^3^	678.9	29.0	0.039	720.8	44.8	0.062
10^2^	69.3	1.1	0.014	73.1	3.6	0.049
10^1^	6.6	1.1	0.147	11.9	5.8	0.483
1	1.8	1.2	0.633	1.7	1.3	0.721
**HPV45**						
10^5^	74,293.3	226.27	0.002	70,117.78	6,557.24	0.094
10^4^	7,392.0	308.60	0.033	7,592.33	424.57	0.056
10^3^	678.4	38.80	0.046	721.83	40.12	0.056
10^2^	70.4	2.26	0.026	75.53	5.31	0.070
10^1^	12.3	2.36	0.153	9.42	5.39	0.572
1	2.7	2.44	0.733	1.71	0.87	0.510

*SD, standard deviation; CV, coefficient of variation.*

### HPV DNA Detection in Clinical Samples and Cell Lines by ddPCR and qPCR

The presence of HPV DNA was investigated by ddPCR and qPCR in CIN specimens (*n* = 45), SiHa, CaSki, HeLa, and HaCaT cell lines. HPV DNA was detected in 40% (18/45) CIN with both assays, whereas an additional 5 CIN, which resulted positive for ddPCR, tested negative in qPCR (Table 2). Specifically, ddPCR detected HPV DNA in 51.1% (23/45) CIN (Table 2). In cell lines, ddPCR/qPCR concordantly indicated the presence of HPV DNA in SiHa, CaSki, and HeLa, whereas HaCaT tested HPV-negative as expected. In addition, qPCR-genotyping indicated that HPV16 and HPV18 types were present in 61.1% (11/18) and 38.9% (7/18) of CIN specimens, respectively, whereas SiHa and CaSki cell lines were HPV16-positive and HeLa cell line resulted HPV18-positive. QPCR-genotyping results were further confirmed by HPV type-specific qPCR employing HPV16 and HPV18 specific primers.

**TABLE 2:** Comparative analysis between droplet digital PCR and quantitative PCR methods, in detection HPV DNA sequences in uterine Cervical Intraepithelial Neoplasia specimens.

**Detection method**	**ddPCR**		
	**Positive**	**Negative**	**Total**
**qPCR**			
Positive	18	0	18
Negative	5	22	27
Total	23	22	45

*ddPCR, Droplet digital PCR; qPCR, Quantitative PCR.*

**FIGURE 6:**
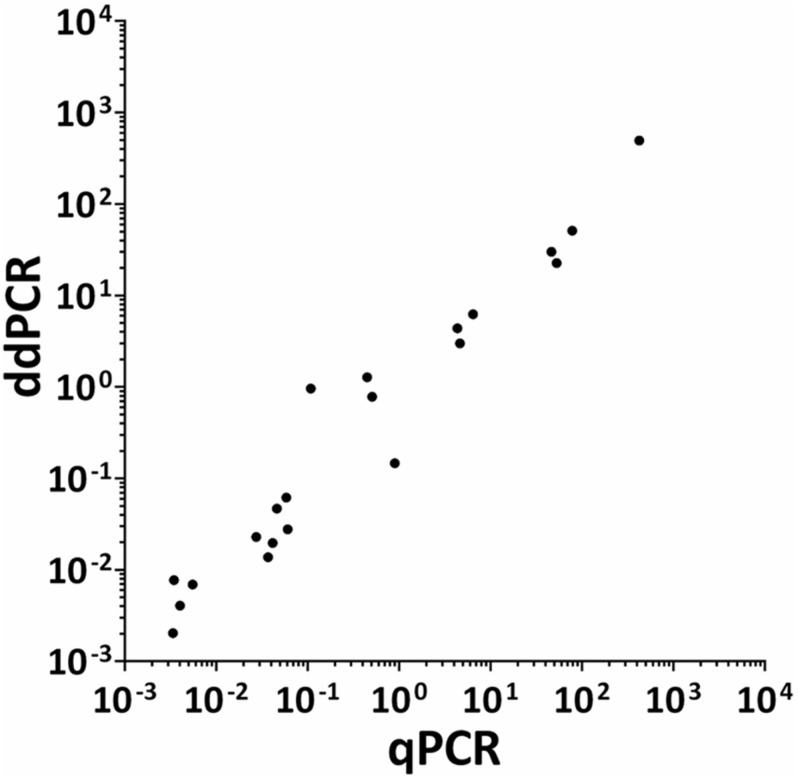
Correlation between ddPCR and qPCR results for HPV viral DNA load in CIN specimens and cervical cancer cell lines. Quantitative results obtained by both ddPCR and qPCR in HPV-positive samples (*n* = 21), including CIN specimens (*n* = 18) and SiHa, CaSKi, and HeLa cervical cancer cell lines, were compared between the two respective assays. Quantitative results are reported as Log 10 of HPV DNA copy/cell. Results from ddPCR and qPCR significantly correlated with a *R*^2^ = 0.9706 and *P* = 0.0001. Linear regression analysis was performed using GraphPad Prism software.

The HPV DNA load in CIN specimens and cell lines was analyzed using both ddPCR and qPCR. The mean HPV DNA loads determined by ddPCR were 4.87 copy/cell (range 0.002–51.02 copy/cell) and 3.81 copy/cell (range 0.002–51.02 copy/cell) in CIN found to be HPV-positive with respective methods (*n* = 18) and with ddPCR alone (*n* = 23), respectively. Quantitative results obtained by qPCR in CIN (*n* = 18) indicate that the mean HPV DNA load was 8.04 copy/cell (range 0.003–78.73 copy/cell). The mean HPV DNA load detected in SiHa and CaSKi cell lines by ddPCR was 3 and 493.9 copy/cell, respectively, whereas HeLa cell line harbored 30 HPV copy/cell. QPCR data indicate that SiHa and CaSki cell lines harbored 4.66 and 424.17 HPV copy/cell, whereas 46.64 HPV copy/cell were detected in HeLa cell line. Quantitative results obtained by ddPCR/qPCR in HPV-positive samples (*n* = 21), including CIN (*n* = 18) and cell lines (*n* = 3) were then compared between the two PCR assays (Figure 6). In the entire group of HPV-positive samples, linear regression analysis indicates good correlation between values, with an *R*^2^ of 0.9706 and a *p* < 0.0001 (Figure 6).

## Discussion

In this study, a reliable ddPCR method was developed to allow the simultaneous detection and quantification of different HPV types in one experimental run employing a wide-range primer set, i.e., GP5^+^/GP6^+^, and the universal fluorescent EvaGreen probe. The need for more analytical HPV-specific techniques in prompted us to set up and to develop this new assay (Sundström et al., 2013). The ddPCR was set up using plasmids containing HPV16/18/11/45 genomes, whereas its validation was performed on CIN specimens and human cell lines. DdPCR quantitative results were compared to those obtained by qPCR on the same specimens/cell lines.

GP5^+^/GP6^+^ primers target the L1 genomic region of HPV16/18/11/45 (Jacobs et al., 1995). These primers have been efficiently used for PCR/qPCR-based HPV studies (de Araujo et al., 2009; Malagutti et al., 2020), making them ideal for transfer to ddPCR. Furthermore, GP5^+^/GP6^+^ primers were selected for their wide range detection potential (Jacobs et al., 1995; de Araujo et al., 2009; Malagutti et al., 2020). Indeed, a number of studies reported that these primers are highly efficient in simultaneously detecting, at different PCR/qPCR conditions, DNAs from HR-HPVs, including HPV16/18/31/33/45 as well as from LR-HPVs, including HPV6/-11 (Jacobs et al., 1995; de Araujo et al., 2009; Malagutti et al., 2020). In our preliminary investigation, GP5^+^ GP6^+^ were tested in ddPCR on: (i) three HR-HPVs, including HPV16 and HPV18, which are considered the most common oncogenic HR-HPVs; (ii) HPV45, which has been frequently found in cervical carcinomas and others malignancies of the anogenital/upper-respiratory tract (Gheit, 2019); (iii) one LR-HPV, HPV11, which has been related to benign genital warts or condylomata acuminata (Gheit, 2019). Notably, HPV16/18/45/11 are all targeted by both quadrivalent and non-avalent vaccines (Ciavattini et al., 2020).

ddPCR performance maximization indicated 225 nM as the optimal primer concentration and a low annealing temperature, 48°C, resulted in both cases as the best amplitude separation between positive and negative droplets (Wu et al., 2018). These results indicate, for the first time, that GP5^+^/GP6^+^ can be applied to ddPCR-based assays. The ddPCR was then tested on circular and restriction enzyme-digested linearized plasmids carrying the HPV16 genome (Yang et al., 2018). Plasmid linearization strongly increased ddPCR efficiency, highlighting the prerequisite of plasmid linearization (Yang et al., 2018). Previous studies reported the higher ddPCR-amplification efficiency in digested-linearized plasmid samples compared to undigested-circular DNA (Yang et al., 2018). As Dong et al. (2014) reported, it is relatively easy for the primer/probe and the DNA polymerase to bind a target region of a linear plasmid compared to undigested supercoil plasmid, because of the exposure of the target sequence of the digested plasmid. Further, these authors found that amplification of undigested-circular plasmids led to an under-estimation of the true copy number. Another work reported that in-reaction, digestion of the DNA with restriction enzymes, enhanced the partitioning of DNA into droplets (Stevenson et al., 2020), thereby improving the ddPCR-amplification efficiency. Accordingly, to evaluate the reliability of our ddPCR assay both digested and undigested plasmids were employed. Our data indicate that ddPCR amplicons from circular/episomal controls are almost undetectable. Then, ddPCR tests were performed on linearized plasmids.

DdPCR specificity was evaluated on a plasmid carrying the complete HPV52 genome, which is closely related to HPV16/18/11/45, as a negative control. In fact, although HPV52 presents a consensus sequence for GP5^+^/GP6^+^ primers (De Roda Husman et al., 1995), it has been reported that its detection using these primers is not reliable (Evans et al., 2005; Chan et al., 2006; Hesselink et al., 2008; Clifford et al., 2016). Chan et al. (2006) described that GP5^+^/GP6^+^ exhibited a poor sensitivity for HPV52 arguing that this virus, which should rank second in prevalence among squamous cell cervical carcinoma in Hong Kong, could be missed if GP5^+^/GP6^+^ are used for HPV detection. Likewise, an additional study reported HPV52, only when amplifying samples with SPF10 but not with GP5^+^/GP6^+^ (Hesselink et al., 2008). Furthermore, Clifford et al. (2016) stated that GP5^+^/GP6^+^ might be particularly insensitive to amplify HPV52 variants that are over-represented outside Europe. As expected, no cross-annealing was found, since the plasmid containing HPV52 tested negative (Evans et al., 2005). These data indicate that ddPCR meets the specific requirement for amplifying HPV16/18/11/45 DNAs without cross-reaction. 10-fold plasmid dilutions, from 10^5^ to 10^0^ copy copy/μL, with HPV16/18/11/45 DNAs were performed for the ddPCR-set up tests. It was reasoned that the ddPCR theoretical dynamic range in detecting HPV DNA was no more than six orders of magnitude (Jones et al., 2016). Indeed, it is known that Poisson statistics are invalidated when droplets saturation point is reached, and this occurs when high target concentrations are tested (Tang et al., 2016). We reached amplitude saturation at 10^6^ copy number/reaction of plasmids, thereby invalidating the calculations (Tang et al., 2016). Consistently, for ddPCR sensitivity, the lower detection limit was one copy/reaction. Therefore, although ddPCR had a relatively narrow dynamic range, our assay resulted valuable in quantifying HPV DNA, especially in low template copy-number conditions. The reason is that mixture partitioning into thousands of droplets improves assay sensitivity (Tagliapietra et al., 2020). However, it should be recalled that due its high sensitivity, an increase in false-positive rates could occur (Zhang et al., 2019). In this study, in order to circumvent these potential drawbacks, negative controls were included in ddPCR runs at all times. No false-positives were found, as none of the controls tested positive, indicating high method reliability (Wu et al., 2018). As several studies reported that ddPCR can quantify few copies of viral DNA (Yang et al., 2018), ddPCR has been progressively used to study a wide range of viruses (Yang et al., 2017, 2018; Dong et al., 2019), including HPV (Biron et al., 2016; Jeannot et al., 2016; Lillsunde Larsson and Helenius, 2017). Indeed, to the best of our knowledge, three previous works reported on HPV DNA detection/quantification employing ddPCR methods (Biron et al., 2016; Jeannot et al., 2016; Lillsunde Larsson and Helenius, 2017). However, these studies are addressed to the identification of a single specific HPV type, each time. These reported protocols require a large number of experimental runs to identify one specific HPV type, as HPV type specific primer sets and TaqMan probes are employed in each experiment (Biron et al., 2016; Jeannot et al., 2016; Lillsunde Larsson and Helenius, 2017). These approaches are time consuming and with high costs for the detection of HPV DNA, which is far from a laboratory routine. It is important to point out that our study is the first investigation reporting on the development of a ddPCR assay, which is specifically focused on the simultaneous detection of four different HPV types (HPV16/18/45/11) in one ddPCR run, employing a wide-range primer set, i.e., GP5^+^/GP6^+^, and EvaGreen dye, which is an universal fluorescent probe for qPCR/ddPCR. We selected GP5^+^/GP6^+^ primer set for its HPV-type wide range detection potential by PCR/qPCR, which has been documented in many studies, including our investigations (Jacobs et al., 1995; de Araujo et al., 2009; Malagutti et al., 2020). Our methodological approach differs from previous HPV ddPCR assays, it improves the viral DNA detection rate. Indeed, we can simultaneously detect four different HPV types in one experimental run, thereby decreasing time and cost efforts. On the other hand, the selection of a wide-range primer set alongside universal EvaGreen dye may limit the potential of our assay, as the HPV type cannot be identified in one experiment. However, our assay can be employed as a first screening approach for the detection/quantification of HPV DNA. Then, only HPV-positive samples can undergo type-specific PCR, thereby avoiding additional/unnecessary HPV-genotyping experiments on HPV-negative samples.

We demonstrated GP5^+^/GP6^+^ primers can potentially detect HPV16/18/45/11. Moreover, previous PCR/qPCR data indicate that these primers can detect additional HPVs, such as HPV6/31/33/58, as well as other HPV types (Jacobs et al., 1995; de Araujo et al., 2009). Further studies are needed to assess whether our ddPCR method might be extended for the detection of additional HPVs.

DdPCR accuracy indicated a good linear correlation between measured and theoretical values among the four 10-fold diluted plasmids, with a *R*^2^ ranging 0.999–1, thus indicating that the ddPCR can quantify different HPV DNAs with high accuracy (Yang et al., 2017, 2018; Dong et al., 2019). To evaluate assay repeatability and reproducibility, each 10-fold diluted plasmid was ddPCR-amplified and intra- and inter-assay CV were determined (Liu et al., 2019). Apart from a few exceptions, the ddPCR showed relatively low intra-/inter-assay CV, as the variance among replicates approached the mean over replicates (Brunetto et al., 2014; Liu et al., 2019). Results indicate that our ddPCR can provide a repeatable and reproducible quantification of HPV DNA, whereas it can be potentially used for patient longitudinal monitoring, given that HPV clearance is recorded over time (Thulin Hedberg et al., 2018).

Following ddPCR set up, as first approach on biological/clinical samples, we extended the assay on restriction enzyme-undigested DNA isolated from 45 CIN tissues and 4 human cells lines, including 3 HPV-positive cervical cancer cell lines. To this end, we considered a Cancer Genome Atlas study showing that HPV integration occurs in >80% of HPV-positive cervical cancers. Of these, 76% of HPV16-positive samples have integrated HPV, whereas integration is evident in all HPV18-positive samples (McBride and Warburton, 2017). During persist infection of HPV, integration events could often be detected, which are well known to cause genome instability, abnormal gene expression in cells. In another study, the integration ratios of HPV infection were between 32 and 77%, for CINI-III and cervical cancer, respectively (Li et al., 2019). DdPCR results were then compared to those obtained in qPCR using the same GP5^+^/GP6^+^ primers. Although 40% of CIN resulted HPV-positive with both assays, the ddPCR detected HPV DNA in 5 CINs that the qPCR had failed to detect, indicating a high detection rate for our new assay. As expected, both assays indicated that the cervical cancer cell lines SiHa, CaSki, and HeLa were positive for HPV (Ostrowska et al., 2011; Diao et al., 2015), whereas the human skin cell line HaCaT tested HPV-negative (Ram et al., 2018). When comparing ddPCR and qPCR, HPV DNA loads, determined in CIN/cervical cancer cell lines resulting HPV-positive with both assays, were well correlated (*R*^2^ = 0.9706). These qualitative/quantitative data are in agreement with previous studies on CIN lesions (Andersson et al., 2005; Comar et al., 2011b; Cerasuolo et al., 2017) and cervical cancer cell lines (Ostrowska et al., 2011; Diao et al., 2015), thereby underling the robustness of this ddPCR. HPV genotyping indicated that CINs were mainly positive for HPV16, followed by HPV18. Our data are in agreement with previous investigations indicating that HPV16 was the most common genotype in uterine cervical precancerous lesions, followed by HPV 18 (Shoja et al., 2019). However, other studies reported additional HPVs, including HPV 6/11/31/33/45, although at lower prevalence (Ogembo et al., 2015; Shoja et al., 2019). In our study, the small sample size may explain the lack of detection of additional HPVs. HPV16 was detected in SiHa and CaSki cell lines, while HeLa tested HPV18-positive (Wilson, 2013; Ram et al., 2018). These results confirm the pivotal role of oncogenic HPV16/18, in cervical carcinogenesis (Tommasino, 2014). In summary, our data indicate that ddPCR is a valuable technique in quantifying HPV DNA in CIN specimens and cervical cancer cell lines (Biron et al., 2016; Lillsunde Larsson and Helenius, 2017).

The ddPCR method described in this pilot study may be potentially helpful for clinical purposes. Indeed, our ddPCR method might be of interest for patient management since it will allow, employing only one ddPCR run, to detect few copies of HPV DNA in clinical samples. Our primary aim of this investigation was the set up of this assay, which increases the sensitive HPV-related cancer-recognition methods. Indeed, undetectable low-copy HPV DNA could be an indication of integration into the human genome, which can be one of the carcinogenesis steps (Arbyn et al., 2012). On this ground, an accurate estimation of HPV DNA load could be relevant in the patient management after primary diagnosis of HPV-related lesions. DdPCR can be therefore employed to verify if a recurrence occurs, following treatment of CIN and/or cervical cancer, after the identification of HPV genotypes during the first prophylaxis. Our ddPCR method could be used in other HPV-related cancers as well, such as oropharyngeal cancer, where access to the anatomical regions with reported lesions might be difficult. The viral persistence and recurrence identification might be hampered even after tumor removal. In this context, the detection of few copies of HPV could be crucial during patient management/follow-up, therefore underlying the clinical significance of the ddPCR as prognostic tool.

In conclusion, in this study, the ddPCR assay exhibited high sensitivity, accuracy and specificity in quantifying HPV DNA sequences. In our experimental conditions, the method is repeatable and reproducible. The robustness of the ddPCR was also demonstrated through assay validation in CIN specimens and human cell lines. This ddPCR assay is a promising method that offers the potential to simultaneously quantify viral DNAs from different HPV types with a high analytical approach, thereby improving clinical applications, such as patient management after primary diagnosis of HPV-related lesions with HPV-type specific assays.

## Data Availability Statement

The original contributions presented in the study are included in the article/supplementary material, further inquiries can be directed to the corresponding authors.

## Ethics Statement

The studies involving human participants were reviewed and approved by the University Hospital of Ferrara Ethical Committee. The patients/participants provided their written informed consent to participate in this study.

## Author Contributions

JR and FM: conceptualization. JR, CM, LO-G, and CL: data curation, formal analysis, software, validation, and visualization. JR, MT, and FM: funding acquisition. JR, CM, LO-G, CL, and MI: investigation. JR and LO-G: methodology and roles, writing – original draft. MT and FM: project administration, resources, supervision, and writing – review and editing the final version. All authors contributed to the study conception and design.

## Conflict of Interest

The authors declare that the research was conducted in the absence of any commercial or financial relationships that could be construed as a potential conflict of interest.
